# Essential Oil of *Rosmarinus officinalis* L.: Phytochemical Composition, Antioxidant and Antibacterial Activity Against Multidrug-Resistant Strains Isolated from Cucumbers

**DOI:** 10.3390/molecules31203669

**Published:** 2026-10-14

**Authors:** Adriana Hernández-García, Alfredo Madariaga-Navarrete, Nallely Rivero-Perez, Cesar Uriel López-Palestina, Alma Delia Hernández-Fuentes, José Esteban Aparicio-Burgos, Armando Peláez-Acero, Mariana Saucedo-García, Adrian Zaragoza-Bastida

**Affiliations:** 1 Instituto de Ciencias Agropecuarias, Universidad Autónoma del Estado de Hidalgo, Avenida Universidad 133, Colonia San Miguel Huatengo, Santiago Tulantepec de Lugo Guerrero 43775, Hidalgo, Mexico; he123730@uaeh.edu.mx (A.H.-G.); nallely_rivero@uaeh.edu.mx (N.R.-P.); cesar_lopez@uaeh.edu.mx (C.U.L.-P.); almah@uaeh.edu.mx (A.D.H.-F.); pelaeza@uaeh.edu.mx (A.P.-A.); saucedo@uaeh.edu.mx (M.S.-G.); 2 Escuela Superior de Apan, Universidad Autonoma del Estado de Hidalgo, Carretera Apan-Calpulalpan, Km 8, Chimalpa Tlalayote, Apan 43920, Hidalgo, Mexico; jose_aparicio@uaeh.edu.mx

**Keywords:** essential oil, α-pinene, antibacterial activity, multidrug-resistant bacteria, bactericide, food safety

## Abstract

The cucumber (*Cucumis sativus* L.) is eaten raw or lightly processed, which makes it a potential vehicle for foodborne bacteria; the spread of resistant strains has renewed interest in plant-derived control agents. This work characterized the phytochemical profile of *Rosmarinus officinalis* essential oil (REO) and assessed its antioxidant capacity and its antibacterial activity against multidrug-resistant (MDR) strains of *Escherichia coli* and *Staphylococcus aureus* isolated from cucumber fruits. REO was obtained by steam distillation in a Clevenger-type apparatus (yield 1.3%). Gas chromatography coupled with mass spectrometry (GC-MS) identified 21 compounds, among which α-pinene (32.53%), 1,8-cineole (20.73%), and verbenone (6.80%) predominated. Antioxidant activity was 49.60 ± 0.69 µM TE/mL REO for the 2,2′-azino-bis (3-ethylbenzothiazoline-6-sulfonic acid) (ABTS) radical and 13.15 ± 0.51 µM TE/mL REO for the 2,2-diphenyl-1-picrylhydrazyl (DPPH) radical, with IC_50_ values of 24.46 ± 2.11 and 56.43 ± 1.86 mg/mL, respectively. The minimum inhibitory concentration (MIC) ranged from 0.19 to 3.12 mg/mL and the minimum bactericidal concentration (MBC) from 0.39 to 6.25 mg/mL. These results highlight REO’s in vitro bactericidal potential and support its evaluation as a component of disinfectant formulations or edible coatings for cucumber.

## 1. Introduction

The cucumber (*Cucumis sativus* L.) ranks among the most widely consumed vegetables worldwide, both fresh and in minimally processed preparations. Precisely because it rarely undergoes a thermal step before reaching the table, it can carry bacteria of public health significance [1]. Contamination may occur at any link in the chain, from the field to the point of sale [2], where irrigation water of poor microbiological quality and poor handling hygiene serve as the main entry routes [3,4,5].

Among the bacteria most frequently recovered from fruits and vegetables are *Escherichia coli* and species of the genus *Staphylococcus*. *E. coli* is interpreted as an indicator of fecal contamination, whereas the presence of *Staphylococcus* points to handling deficiencies and compromises food safety [1,6]. The situation is more serious when these isolates are multidrug-resistant (MDR) since fresh produce then becomes a route of human exposure and of dissemination along the food chain. Such a scenario has already been documented in vegetables marketed in Mexico: every isolate was MDR to the antibiotics tested, with multiple antibiotic resistance index (MARI) values between 0.42 and 1.00 [7]. MDR strains represent a growing public health problem, because they narrow the therapeutic options available and persist across a wide range of environments [8,9].

Antimicrobial resistance now ranks among the leading threats to global health, and this pressure has redirected research toward new ways to control pathogenic and MDR bacteria [10]. Essential oils are among the most explored natural alternatives to synthetic antimicrobials, and their antibacterial effects involve several simultaneous mechanisms, including disruption of the cytoplasmic membrane, increased permeability, and interference with the metabolic processes bacteria require to grow [9,11]. A prominent example among aromatic species with recognized biological potential is rosemary (*Rosmarinus officinalis* L., syn. *Salvia rosmarinus Spenn*.), a member of the Lamiaceae family with a long record of use in the food, pharmaceutical, and cosmetics industries [12]. Rosemary essential oil (REO) shows antioxidant and antibacterial activity, attributed to its terpene content, particularly monoterpenes [13,14].

REO composition can vary by geographic origin, soil and climate, the plant’s phenological stage, and the extraction method used. The constituents reported most often are α-pinene, 1,8-cineole, camphor, borneol, and verbenone, all of them linked to antioxidant and antibacterial activity [15].

Antioxidant activity in essential oils is commonly evaluated with the 2,2′-azino-bis(3-ethylbenzothiazoline-6-sulfonic acid) (ABTS) and 2,2-diphenyl-1-picrylhydrazyl (DPPH) radicals, although several studies report lower capacity values with DPPH than with ABTS. This difference can be attributed to the DPPH radical’s structural characteristics. Its reactive center, located on a nitrogen atom, is surrounded by three bulky aromatic groups (two phenyls and one picryl); while these contribute to the radical’s stability, they also create steric hindrance. Consequently, antioxidant molecules have limited access to the reactive site, reducing the reaction rate and, in turn, the antioxidant activity values determined by this method. In contrast, the ABTS radical possesses a more accessible reactive center, which facilitates interaction with a wider range of antioxidant compounds and generally yields higher activity values [16,17]. Radical neutralization occurs through electron transfer and hydrogen atom transfer [18]. Both mechanisms can act simultaneously, and solvent type, reaction medium, and the structural and physicochemical characteristics of the antioxidants affect their activity [19]. Taken together, these factors determine the antioxidant values obtained with each method.

The antioxidant potential of REO also depends on the structure of its main constituents. α-Pinene, a hydrocarbon monoterpene, shows moderate antioxidant activity because the activated allylic hydrogens of the methylene group adjacent to the double bond favor hydrogen donation to a free radical [20,21]. In contrast, 1,8-cineole, a cyclic monoterpene ether, has lower hydrogen-donating ability than α-pinene because the oxygen atom in its bicyclic structure limits its antioxidant capacity [22]. Verbenone, in turn, has a ketone group and a carbon–carbon double bond, features associated with a greater ability to neutralize free radicals [23,24]. According to Hcini et al., the antioxidant activity of REO can be attributed to compounds such as α-pinene, 1,8-cineole, α-terpineol, camphor, borneol, and camphene [25]. Nevertheless, this activity may vary with the phytochemical composition and with synergistic and antagonistic interactions among the components [26].

Beyond its antioxidant activity, α-pinene has attracted particular interest for its antibacterial action because it compromises both bacterial cell structure and function [16]. In fact, the antioxidant and antibacterial activities of essential oils are often interrelated since both depend on the presence and relative abundance of terpenic compounds. Monoterpenes such as α-pinene and 1,8-cineole, along with α-terpineol, have been reported to contribute to free-radical neutralization by donating electrons or hydrogen atoms and to exert antimicrobial effects by disrupting bacterial membrane integrity and interfering with cellular metabolism [15,16]. Damage to the bacterial cell wall and membrane is one of the most frequently described mechanisms for plant-derived compounds; for essential oils, it is generally attributed to the interaction of their hydrophobic components with the membrane, which alters its permeability and potential and causes the release of intracellular constituents. However, this effect rarely acts in isolation. In pure compounds such as eugenol, membrane damage is accompanied by increased reactive oxygen species (ROS), pointing to a broader oxidative antibacterial mechanism [27,28]. Consistent with this, REO increased intracellular ROS production in *Staphylococcus aureus* in a dose-dependent manner and decreased catalase activity, a key bacterial defense against oxidative stress, suggesting that oxidative stress-mediated toxicity contributes to its antibacterial action [29].

Consequently, variations in the phytochemical composition of REO may influence both its antioxidant capacity and its antibacterial efficacy. Several studies have shown that essential oils with higher concentrations of these monoterpenes exhibit stronger antioxidant and antimicrobial activities, which suggests that antioxidant capacity can serve as an indirect indicator of their biological potential as antimicrobial agents [30]. In rosemary, this relationship is evident at the chemotype level, since, of two Algerian oils, the one richer in oxygenated monoterpenes (mainly 1,8-cineole and borneol) showed stronger radical scavenging and higher efficacy against *Staphylococcus aureus* and *Klebsiella pneumoniae*. However, this effect cannot be attributed to a single compound, as 1,8-cineole alone is poorly reactive toward free radicals and the overall antioxidant effect appears to depend on synergy with minor constituents [31].

The biological properties of REO are well documented, yet its performance against MDR bacteria recovered from fresh fruits and vegetables has rarely been tested, and fewer studies still have examined its chemical composition, antioxidant capacity, and antibacterial activity together in the same set of isolates. Filling this gap would clarify how far REO can serve as a source of bioactive compounds against bacteria important to food safety.

Therefore, this research aimed to characterize the phytochemical composition of REO and evaluate its antioxidant and antibacterial activities against MDR strains of *Escherichia coli* and *Staphylococcus aureus* isolated from cucumber fruits.

## 2. Results and Discussion

### 2.1. Essential Oil Extraction Yield from R. officinalis

The REO extraction yield was 1.3%, within the essential oil content range of 0.5–2.5% reported for *R. officinalis* by Flores-Villa et al. [12]. This is also close to the 1.2% obtained by Boutekedjiret et al. through steam distillation of rosemary from the Bibans region of Algeria [17]. However, the same authors obtained a yield of 0.44% using hydrodistillation, demonstrating how the extraction method influences oil recovery under the conditions evaluated. Therefore, the numerical similarity to the yield reported in this study should be interpreted by considering both the extraction method and the basis used to calculate the percentage yield, before attributing the differences to the characteristics of the plant material.

In addition to the extraction method, the plant organ used is important for interpreting the differences between studies. The leaves of *R. officinalis* contain glandular structures that produce and accumulate essential oil [18,19]. However, Boutekedjiret et al. [17] did not specify the organ used, limiting comparison. Furthermore, the phenological stage and growing conditions such as climate, precipitation, and soil characteristics can influence essential oil content [20,21]. Therefore, the yield is consistent with values reported for this species, although differences between studies cannot be attributed solely to the extraction method.

### 2.2. Phytochemical Composition of the Essential Oil of R. officinalis

A total of 21 compounds were identified in REO, accounting for 98.38% of the total identified composition (Table 1). The major compounds were α-pinene (32.53%), 1,8-cineole (20.73%), and verbenone (6.80%), which together accounted for 60.06% of the essential oil. Oxygenated monoterpenes constituted the predominant fraction (49.59%), followed by hydrocarbon monoterpenes (45.68%) and hydrocarbon sesquiterpenes (3.11%).

Several chemotypes have been described in *R. officinalis* based on the relative percentages of major constituents [14,32], with the α-pinene chemotype characterized by α-pinene content >20%. Accordingly, the essential oil evaluated in this study corresponds to the α-Pinene was its major constituent (32.53%), followed by 1,8-cineole (20.73%) [33]. α-Pinene is a structural isomer of pinene (C_10_H_16_), a bicyclic hydrocarbon terpenoid with a double bond, and belongs to the monoterpene family. It exists in two enantiomeric forms, (+) and (−), and has been extensively studied for its biological activities as an anti-inflammatory, anticarcinogenic, antispasmodic, antioxidant, antiviral, and antimicrobial agent [15].

Quyen and Quoc reported similar results, identifying α-pinene (33.76%), 1,8-cineole (18.47%), and (−)-verbenone (6.11%) as the major compounds in REO from Vietnam [22]. Furthermore, Mollova et al. reported that the main compounds identified in REO from rosemary of Iranian origin cultivated in Bulgaria were α-pinene (51.53%), verbenone (1.13%), 1,8-cineole (14.24%), camphor (5.05%), bornyl acetate (4.05%), and borneol (2.63%) [23]. These results suggest that α-pinene is one of the predominant compounds in REO; however, the essential oil’s phytochemical composition may vary by plant origin.

Previous studies have reported differences in the phytochemical profile of REO associated with cultivation type. In this regard, Sakar et al. analyzed the essential oil of cultivated and wild plants from Morocco, observing that the major compounds were 1,8-cineole (32.48%), camphor (18.79%), and α-pinene (14.07%) in cultivated plants, whereas in wild plants, 1,8-cineole (40.72%), α-pinene (26.20%), and camphor (19.66%) predominated [24]. These results show that compound variability in REO can change even within a single geographic region.

Similarly, Micić et al. reported that in oils of Serbian and Russian origin, α-pinene was the main component (23.0% and 17.76%), followed by 1,8-cineole (17.79% and 23.40%) and camphor (14.39% and 17.17%) [13]. Taken together, these findings indicate that the phytochemical composition of *R. officinalis* essential oils is influenced by various factors, including geographic origin, soil characteristics, environmental conditions, harvest season, the plant’s chemotype, and the extraction method used [25,26,34]. Therefore, these variations can significantly influence the biological activity of essential oils [35].

### 2.3. Assessment of Antioxidant Activity

The antioxidant capacity of REO was 49.60 ± 0.69 and 13.15 ± 0.51 µM Trolox equivalents (TE)/mL in the ABTS and DPPH assays, respectively. The DPPH value was 73.49% lower than the ABTS value, indicating that REO’s antioxidant response depends on the method used. However, Ben Akacha et al. between the two assays have been reported for rosemary essential oils [33], suggesting that the antioxidant activity determined may be influenced by the physicochemical characteristics of the radicals used, as well as by the oil’s phytochemical composition. Similarly, Ojeda-Sana et al. [30] noted that variations in the antioxidant activity of rosemary essential oils relate to differences in their phytochemical composition and the proportions of their main compounds.

The antioxidant activity reported in the present study may be partly attributed to α-pinene (32.53%), 1,8-cineole (20.73%), and verbenone (6.80%), which together account for 60.06% of the oil’s total composition. Huang et al. [33] reported that rosemary essential oils with higher verbenone concentrations had greater antioxidant activity, associated with a greater capacity to reduce ROS. Similarly, Wang et al. [36] indicated that REO exhibited greater antioxidant activity than its individual compounds, including α-pinene and 1,8-cineole, suggesting that the antioxidant effect of REO depends not only on its individual components but also on synergistic interactions among the phytochemical compounds. Therefore, the antioxidant activity of the REO evaluated was probably influenced by its major compounds, as well as by synergistic, additive, and antagonistic interactions among the different compounds in the oil.

REO exhibited IC_50_ values of 24.46 ± 2.11 mg/mL for ABTS and 56.43 ± 1.86 mg/mL for DPPH (mean ± standard deviation). Lower IC_50_ values indicate greater antioxidant capacity because less sample is required to neutralize 50% of the free radicals [31]. Consistent with the results expressed as Trolox equivalents, the lower IC_50_ value obtained with ABTS indicates greater REO antioxidant activity against this radical. These results suggest that REO exhibits moderate antioxidant activity, which may be attributable to both its phytochemical composition and interactions among its components. Therefore, considering the biological activities reported for the major compounds identified in REO, its antibacterial potential against reference strains and MDR strains was evaluated.

### 2.4. Evaluation (In Vitro) of Antibacterial Activity

#### Determination of the Minimum Inhibitory Concentration (MIC) and Minimum Bactericidal Concentration (MBC)

The in vitro evaluation of REO’s antibacterial activity was performed by determining the MIC and MBC against MDR strains of *Escherichia coli* and *Staphylococcus aureus*. Table 2 presents the results.

Despite their differing resistance profiles to conventional antibacterials, REO inhibited the growth of all strains evaluated. MIC values ranged from 0.19 to 3.12 mg/mL, while MBC values ranged from 0.39 to 6.25 mg/mL. Furthermore, MIC and MBC values differed significantly (*p* ≤ 0.05) among the strains evaluated. REO showed the strongest inhibitory activity against the *S. aureus* strains (M_19_B_1M_ and M_3_B_2M_), with MIC values of 0.19 mg/mL. In particular, the *S. aureus* strain (M_3_B_2M_) had the lowest MBC (0.39 mg/mL).

In contrast, the *E. coli* strain (M_14_B_1MK_) showed the highest MIC (3.12 mg/mL) and MBC values (6.25 mg/mL). Meanwhile, the reference strains *E. coli* ATCC ^35218^ and *S. aureus* ATCC ^6538^ had MIC values of 0.39 mg/mL and MBC values of 0.78 mg/mL.

In addition, we determined the essential oil’s effect based on the MBC to MIC ratio. The results indicated a bactericidal effect against all *E. coli* strains. However, the *S. aureus* strains showed a different pattern: three of the four strains evaluated (M_6_B_1T_, M_3_B_2M_, M_10_B_1T_) were bactericidal, whereas M_19_B_1M_ was bacteriostatic. This latter strain is of interest because it exhibited the highest multiple antibiotic resistance index (MARI); however, REO demonstrated activity against this MDR strain, highlighting its potential as an alternative for controlling bacteria resistant to antibacterial agents.

No significant correlation was found between MARI and the MIC (ρ = 0.130, *p* = 0.758) or MBC (ρ = 0.304, *p* = 0.464) of REO. Strains with greater antibiotic resistance did not consistently require higher oil concentrations to inhibit growth or achieve a bactericidal effect. Ebani et al. [37], described a similar observation: an *E. coli* isolate that was non-susceptible to all antibiotics tested remained susceptible to five essential oils. Their findings are consistent with the activity of REO against the multidrug-resistant isolates evaluated in this study.

REO showed greater activity against *S. aureus* strains (MIC ranging from 0.19 to 0.78 mg/mL) than against *E. coli* isolates (MIC ranging from 0.39 to 3.12 mg/mL), consistent with previous studies reporting greater inhibitory activity of essential oils against Gram-positive bacteria. Jiang et al. [11] reported the lowest MIC for REO against *S. aureus* MRSA 53 (0.03% *v*/*v*) and the highest against *E. coli* ATCC 8739 (0.3% *v*/*v*), while Puvača et al. [9] reported activity against *S. aureus* ATCC 25923 (MIC of 1.40 mg/mL), with no effect on *E. coli*. In contrast, REO also inhibited MDR *E. coli* isolates. This difference has been primarily attributed to structural differences between the two bacterial groups. Unlike Gram-positive bacteria, Gram-negative bacteria have an outer membrane that limits penetration of hydrophobic compounds, reducing the inhibitory effect of essential oil components [38].

In addition, antibacterial activity varied among the *E. coli* strains evaluated. In particular, the lower activity of REO against strain M_14_B_1MK_ could be associated with intrinsic or acquired resistance mechanisms [39]. However, the oil exerted both inhibitory and bactericidal effects on this strain, which is noteworthy given its resistance profile.

Several authors have noted that the phytochemical composition of REO varies and that its antibacterial activity depends on the presence and proportion of its bioactive compounds [11,32]. α-Pinene, the major compound in REO in this study, is a bicyclic hydrocarbon monoterpene without polar groups; this characteristic is associated with high lipophilicity and, according to the literature, allows it to integrate into the cytoplasmic membrane, increase permeability, and cause leakage of cellular contents [28]. Jiang et al. reported an MIC of 0.3% against *S. aureus* MRSA 53 and 1.0% against *E. coli* [11]. In addition, it has been reported to modulate resistance mechanisms by inhibiting efflux pumps [40]. Because 1,8-cineole is a monoterpene ether with lower lipophilicity, its action has been reported to be limited mainly to Gram-positive bacteria [11]. However, Ben Akacha et al. [41] evaluated 1,8-cineole against *S. aureus* and *E. coli* and reported MIC values of 0.45 ± 0.14 mg/mL for both strains. These findings highlight the antibacterial potential of this oxygenated monoterpene and may contribute to the antimicrobial activity of REO observed in this study. Oxygenated monoterpenes such as verbenone have been reported to alter bacterial membranes, increase permeability, and interfere with enzymatic systems [33]. Al-Tarawneh attributed this activity to the high content of oxygenated monoterpenes in an REO of the verbenone chemotype (27.14%, compared with 6.80% in the present study), which exhibited an MIC of 0.9 mg/mL and inhibition zones of 19.0 and 20.0 mm against *S. aureus* and *E. coli* [38].

It has been reported that the mechanism of action of essential oils primarily involves altering the bacterial membrane; this is the case in the study by Gayosso-San Juan et al. [42], who demonstrated that clove essential oil (*Syzygium aromaticum*), with a high eugenol content (73.9%), disrupts the cell membrane of MDR bacteria. The extent of damage was assessed by quantifying proteins and nucleic acids in the different treatments; increases in the extracellular medium relative to the control confirmed loss of membrane integrity and leakage of intracellular components. Consistent with the above, the antibacterial activity of REO is unlikely to be attributable to a single constituent and probably reflects the combined action of hydrocarbon and oxygenated monoterpenes, whose ratio may influence the effect observed on Gram-positive and Gram-negative bacteria; however, this study did not evaluate the individual contribution of each compound. In the present study, REO showed inhibitory and bactericidal activity in vitro against MDR bacteria of public health importance. These in vitro results suggest its potential as a source of bioactive compounds for developing sustainable and effective antibacterial alternatives. In this context, previous studies have evaluated essential oils (thyme, oregano, cinnamon, and clove) as alternatives to chemical sanitizers in postharvest washing processes for fresh produce [43]. Researchers have incorporated REO into emulsions, nanoencapsulated systems, and edible coatings for food preservation [44]. These precedents support evaluating REO as a potential sanitizing agent during the postharvest handling of fresh produce such as cucumber.

## 3. Materials and Methods

### 3.1. Collection and Identification of Plant Material

The aerial parts of *R. officinalis* L. were collected in the town of Santa María Nativitas, in the municipality of Cuautepec de Hinojosa, Hidalgo (20°1′48” N, 98°18′36” W), from January to February 2025. The species was identified as *Rosmarinus officinalis* L. (IBUNAM: MEXU: 212821).

### 3.2. Extraction of Essential Oil

Shade-dried leaves of *R. officinalis* (22 ± 2 °C) were steam-distilled for 3.5 h in a Clevenger-type system using 50 g of plant material in 1 L of distilled water, as described by Toazza et al. [45]. The essential oil obtained was dried with anhydrous sodium sulfate (Na_2_SO_4_, analytical grade, J. T. Baker, Avantor Performance Materials, Ecatepec, Mexico) and stored in amber vials at 4 °C until analysis. Extraction yield was calculated according to Dhouibi et al. [46]:

(1) $$Essential\;Oil\;Yield\;\left(\%\right)=\;\frac{Weight\;of\;the\;harvested\;essential\;oil}{Dry\;weight\;of\;plant\;material}\;\times \;100$$

### 3.3. Gas-Chromatography/Mass-Spectrometry (GC-MS) Analysis

Chromatographic analysis of REO was performed following the methodology described previously [47]. Briefly, the sample (diluted 1:50 in analytical-grade methanol; Meyer, Mexico City, Mexico) was analyzed on an Agilent Technologies 7890A GC (Agilent Technologies, Santa Clara, CA, USA) coupled to a 5975 GC/MSD detector, fitted with an HP-5MS capillary column (30 m × 0.25 mm, 0.25 µm) and using helium as carrier gas (1 mL/min, splitless injection at 260 °C). The oven program was 60 °C for 5 min, ramped at 3 °C/min to 240 °C and held for 10 min; ionization was performed at 70 eV over a scan range of 40–400 *m*/*z*.

Agilent MassHunter software (B.07) was used to identify compounds by comparing the chromatogram mass spectra with the National Institute of Standards and Technology (NIST) database [22]. The Score (Lib) value, which expresses the degree of spectral similarity, was used as a supporting criterion for identification [48]. Relative abundance was expressed as the percentage of each peak area with respect to the total area [22].

### 3.4. Evaluation of the Antioxidant Activity of the Essential Oil

All reagents used were of analytical grade. A stock solution of REO (50 mg in 10 mL of methanol) was used for both the ABTS and DPPH assays. The ABTS assay was carried out as described by López-Palestina et al. [49], with minor modifications. The ABTS•+ radical was generated from 7 mM ABTS (Sigma-Aldrich, Toluca, Mexico) and 2.45 mM potassium persulfate (Meyer, Mexico City, Mexico) after 16 h of stirring in the dark; 100 µL of the REO solution was then added to 3 mL of the stabilized radical and, after 6 min in the dark, absorbance was read at 734 nm (Genesys 150 UV, Thermo Scientific, Waltham, MA, USA).

The DPPH assay was performed as described previously [50], with minor modifications. The REO solution was reacted for 1 h at 4 °C with a methanolic solution of DPPH• (6 × 10^−5^ M; Sigma-Aldrich, Toluca, Mexico), previously stirred for 2 h in the dark, and absorbance was read at 517 nm (Genesys 150 UV, Thermo Scientific, Waltham, MA, USA). Both assays were performed in triplicate and the results were expressed as micromoles of Trolox equivalents (TE) per mL of REO (µM TE/mL REO).

### 3.5. Calculation of IC_50_ Values

The IC_50_ value is defined as the amount of essential oil required to inhibit 50% of the free radicals [51]. It was estimated by simple linear regression of radical scavenging activity (%) against REO concentration [52]. The regression was fitted independently for each replicate, using the concentration ranges of 3.02–96.67 mg/mL for ABTS (R^2^ = 0.983–0.989) and 0.76–96.67 mg/mL for DPPH (R^2^ = 0.961–0.976). In both assays, IC_50_ values fell within the tested range. Results are expressed as mean ± SD (n = 3).

### 3.6. Determination of Minimum Inhibitory Concentration (MIC) and Minimum Bactericidal Concentration (MBC)

The minimum inhibitory concentration (MIC) and minimum bactericidal concentration (MBC) of REO were determined according to the guidelines of the Clinical and Laboratory Standards Institute (CLSI) [53] and previously established protocols [54], with slight modifications.

Two reference strains from the American Type Culture Collection (ATCC) were evaluated: *Escherichia coli* ATCC ^35218^ and *Staphylococcus aureus* ATCC ^6538^. In addition, multidrug-resistant strains of *E. coli* (M_6_B_1MK_, M_14_B_1MK_, M_1_B_1MK_, and Strain_195_) and *S. aureus* (M_19_B_1M_, M_6_B_1T_, M_3_B_2M_, and M_10_B_1T_) were used. These strains were previously isolated from cucumber fruits and characterized at the Bacteriology Laboratory, Institute of Agricultural Sciences (ICAp), Autonomous University of the State of Hidalgo (UAEH). This characterization was performed using the Kirby–Bauer disk diffusion method, following CLSI interpretive criteria. The antimicrobial susceptibility profiles and MARI values presented in Table 3 and Table 4 are included as background information on the strains used.

The bacterial strains were reactivated and cultured in nutrient broth (BD Bioxon, Heidelberg, Germany) at 37 °C for 24 h. The bacterial suspensions were then adjusted to a 0.5 McFarland turbidity standard (Remel, R20421, Lenexa, KS, USA), corresponding to approximately 1 × 10^8^ CFU/mL. REO was emulsified with 0.5% (*v*/*v*) polysorbate 80 (Tween 80^®^) and serially diluted to obtain concentrations ranging from 50 to 0.095 mg/mL. The Tween 80 concentration decreased proportionally with each dilution. The assay was performed in sterile 96-well plates by adding 90 μL of each REO dilution and 10 μL of the bacterial suspension to each well, for a final volume of 100 μL. The plates were incubated at 37 °C for 24 h. Bacterial viability was assessed by adding 20 μL of 0.04% (*w*/*v*) p-iodonitrotetrazolium chloride solution (Sigma-Aldrich 18377, St. Louis, MO, USA) to each well. The plates were then incubated for an additional 30 min at 37 °C with constant shaking and uninoculated broth was used as the sterility control ng (70 rpm). The MIC was defined as the lowest REO concentration that completely inhibited visible bacterial growth, as indicated by the absence of a color change from yellow to pink.

To determine the MBC, 5 μL was taken from each well before adding p-iodonitrotetrazolium and plated on Mueller-Hinton agar (DIBICO^®^, Cuautitlán Izcalli, Mexico). The plates were incubated at 37 °C for 24 h. The MBC was defined as the lowest REO concentration at which no bacterial growth was observed. The MBC/MIC ratio was used to classify the essential oil’s effect as bactericidal (≤4) or bacteriostatic (>4), according to Rangel-López et al. [55].

Kanamycin (≥750 I.U./mg, AppliChem GmbH, Darmstadt, Germany) was used as a positive control at concentrations ranging from 64 to 0.12 μg/mL. Nutrient broth containing 0.5% (*v*/*v*) Tween 80 and no REO, corresponding to the highest surfactant concentration used, served as the vehicle control. The growth and sterility controls consisted of nutrient broth without REO or Tween 80 and nutrient broth without an inoculum. We performed three independent biological replicates on separate occasions using freshly prepared bacterial cultures and REO dilutions. In each replicate, we tested every treatment and control in triplicate.

### 3.7. Statistical Analysis

All experiments were performed in triplicate, and the results are expressed as the mean ± standard deviation. Data were log10-transformed, and normality was confirmed by the Shapiro–Wilk test prior to analysis of variance (ANOVA) and Tukey’s test for mean comparison (*p* ≤ 0.05). The association between MARI and the MIC and MBC values of rosemary essential oil was determined using Spearman’s rank correlation coefficient (ρ) for the eight cucumber-derived isolates and reference strains. All analyses were performed in SAS v.9.4 (Statistical Analysis System, Cary, CA, USA).

## 4. Conclusions

The essential oil of *Rosmarinus officinalis* L. exhibited a phytochemical profile characterized mainly by α-pinene (32.53%), 1,8-cineole (20.73%), and verbenone (6.80%), and showed greater antioxidant activity in the ABTS assay than in the DPPH assay. REO inhibited all the strains evaluated, with MIC values ranging from 0.19 to 3.12 mg/mL and MBC values ranging from 0.39 to 6.25 mg/mL. It showed bactericidal activity against all *E. coli* strains, the *S. aureus* reference strain, and three of the four MDR *S. aureus* isolates; bacteriostatic activity was observed only against strain M_19_B_1M_. Overall, REO showed greater inhibitory activity against *S. aureus* than against *E. coli*. These results demonstrate REO’s antioxidant and in vitro antibacterial potential. However, further studies directly on cucumber fruits are required to evaluate the efficacy, safety, stability, and sensory effects of REO-based disinfectant formulations or edible coatings.

## Figures and Tables

**Table 1:** Phytochemical components identified in the essential oil of *Rosmarinus officinalis*.

No.	Component	RT (min)	Molecular Formula	RA (%)
1	α-Pinene	6.53	C_10_H_16_	32.53
2	Camphene	7.09	C_10_H_16_	4.49
3	β-Pinene	8.07	C_10_H_16_	3.10
4	β-Myrcene	8.72	C_10_H_16_	1.81
5	α-Terpinene	9.74	C_10_H_16_	0.61
6	1,8-Cineole	10.40	C_10_H_18_O	20.73
7	γ-Terpinene	11.53	C_10_H_16_	1.66
8	Terpinolene	12.85	C_10_H_16_	1.49
9	Linalool	13.52	C_10_H_18_O	3.35
10	Chrysanthenone	14.51	C_10_H_14_O	1.42
11	Camphor	15.33	C_10_H_16_O	2.56
12	Borneol	16.44	C_10_H_18_O	3.62
13	Pinocamphone	16.72	C_10_H_16_O	0.87
14	Terpinen-4-ol	16.94	C_10_H_18_O	0.92
15	α-Terpineol	17.63	C_10_H_18_O	2.03
16	(−)-Myrtenol	17.88	C_10_H_16_O	0.55
17	Verbenone	18.52	C_10_H_14_O	6.80
18	Geraniol	20.75	C_10_H_18_O	3.76
19	(−)-Bornyl acetate	21.89	C_12_H_20_O_2_	2.41
20	Geranyl acetate	26.30	C_12_H_20_O_2_	0.56
21	Caryophyllene	27.59	C_15_H_24_	3.11
Total identified				98.38

RT, retention time; RA, relative abundance.

**Table 2:** Minimum inhibitory concentration (MIC) and minimum bactericidal concentration (MBC) of *Rosmarinus officinalis* essential oil against reference bacterial strains and multidrug-resistant strains of *Escherichia coli* and *Staphylococcus aureus*.

Strain ID	MIC REO (mg/mL)	MBC REO (mg/mL)	MBC/MIC	MIC Kn µg/mL	MBC Kn µg/mL
*E. coli* ATCC ^35218^	0.39 ^b^	0.78 ^b^	2.00	1.00 ^d^	2.00 ^d^
M_6_B_1MK_	1.56 ^d^	3.12 ^d^	2.00	0.25 ^b^	0.50 ^b^
M_14_B_1MK_	3.12 ^e^	6.25 ^e^	2.00	1.00 ^d^	2.00 ^d^
M_1_B_1MK_	0.39 ^b^	0.78 ^b^	2.00	2.00 ^e^	4.00 ^e^
Strain_95_	0.39 ^b^	0.78 ^b^	2.00	1.00 ^d^	2.00 ^d^
*S. aureus* ATCC ^6538^	0.39 ^b^	0.78 ^b^	2.00	1.00 ^d^	2.00 ^d^
M_19_B_1M_	0.19 ^a^	0.78 ^b^	4.11	0.25 ^b^	0.50 ^b^
M_6_B_1T_	0.78 ^c^	1.56 ^c^	2.00	0.50 ^c^	1.00 ^c^
M_3_B_2M_	0.19 ^a^	0.39 ^a^	2.05	0.12 ^a^	0.25 ^a^
M_10_B_1T_	0.39 ^b^	1.56 ^c^	4.00	0.12 ^a^	0.25 ^a^

REO, essential oil; Kn, kanamycin; ^a, b, c, d, e^ different letters indicate statistically significant differences among bacteria.

**Table 3:** Antimicrobial susceptibility and multiple antibiotic resistance index (MARI) of the *Escherichia coli* strains used to evaluate antibacterial activity.

Antimicrobial	*Escherichia coli*			
	M_6_B_1MK_	M_14_B_1MK_	M_1_B_1MK_	Strain_195_
Amikacin	(R)	(R)	(R)	(S)
Ampicillin	(R)	(R)	(R)	(R)
Carbenicillin	(R)	(R)	(R)	(R)
Cephalothin	(R)	(R)	(R)	(R)
Cefotaxime	(R)	(R)	(R)	(R)
Ciprofloxacin	(R)	(R)	(R)	(R)
Chloramphenicol	(R)	(R)	(R)	(R)
Gentamicin	(R)	(R)	(S)	(S)
Netilmicin	(R)	(S)	(S)	(S)
Nitrofurantoin	(R)	(R)	(S)	(S)
Norfloxacin	(R)	(R)	(R)	(S)
Sulfamethoxazole/Trimethoprim	(R)	(R)	(S)	(S)
MARI	1.00	0.92	0.67	0.50

S, sensitive; R, resistant; MARI, Multiple Antibiotic Resistance Index.

**Table 4:** Antimicrobial susceptibility and multiple antibiotic resistance index (MARI) of the *Staphylococcus aureus* strains used to evaluate antibacterial activity.

Antimicrobial	*Staphylococcus aureus*			
	M_19_B_1M_	M_3_B_2M_	M_6_B_1T_	M_10_B_1T_
Ampicillin	(R)	(R)	(R)	(R)
Cephalothin	(R)	(R)	(R)	(R)
Cefotaxime	(R)	(R)	(R)	(R)
Ciprofloxacin	(R)	(S)	(S)	(S)
Clindamycin	(R)	(R)	(R)	(R)
Dicloxacillin	(R)	(R)	(R)	(R)
Erythromycin	(R)	(R)	(R)	(R)
Gentamicin	(R)	(S)	(R)	(S)
Penicillin	(R)	(R)	(R)	(R)
Tetracycline	(R)	(R)	(S)	(S)
Sulfamethoxazole/ Trimethoprim	(R)	(R)	(S)	(S)
Vancomycin	(R)	(R)	(R)	(R)
MARI	1.00	0.83	0.75	0.67

S, sensitive; R, resistant; MARI, Multiple Antibiotic Resistance Index.

## Data Availability

The data generated and analyzed in this study may be obtained from the corresponding author on reasonable request. These data are not publicly accessible, as they are subject to intellectual property protection.
